# Análise dos achados microbiológicos identificados na superfície dos pinos de fixadores externos comparando pinos de aço com pinos revestidos por hidroxiapatita

**DOI:** 10.1055/s-0045-1809519

**Published:** 2025-06-23

**Authors:** Cristhopher Stoffel, Honório Octávio Cuadro Peixoto, Felipe Kowaleski dos Santos, Pedro Afonso Keller Licks, Fernando Baldy dos Reis, Mauro José Costa Salles

**Affiliations:** 1 Instituto de Ortopedia e Traumatologia, Hospital São Vicente de Paulo, Passo Fundo, RS, Brasil; 2 Departamento de Ortopedia e Traumatologia, Escola Paulista de Medicina, Universidade Federal de São Paulo, São Paulo, SP, Brasil; 3 Disciplina de Infectologia, Grupo de Infecção Musculoesquelética, Escola Paulista de Medicina, Universidade Federal de São Paulo, São Paulo, SP, Brasil

**Keywords:** fios ortopédicos, fixadores externos, hidroxiapatita, infecções, pinos ortopédicos, bone nails, bone wires, external fixators, hydroxyapatite, infections

## Abstract

**Objetivo:**

Comparar as taxas de recuperação microbiana e os tipos de microrganismos identificados na superfície dos pinos de aço inoxidável (PAIs) e nos pinos revestidos com hidroxiapatita (PHAs) de fixadores externos (FEs).

**Métodos:**

Este estudo de coorte prospectiva de intervenção, não randomizado, multicêntrico, comparative foi realizado entre abril de 2018 e outubro de 2021, com 44 pacientes tratados com FE, 33 dos quais receberam PAIs e 11 receberam PHAs. Foram coletados e enviados para análise microbiológica dois pinos de cada paciente, o de melhor e o de pior aspecto clínico conforme a classificação de Maz-Oxford-Nuffield (MON), de forma asséptica.

**Resultados:**

A taxa de infecção (TI) superficial global foi de 52,3% (23 de 44 pacientes), sendo 45,5% (5 de 11 pacientes) entre pacientes que receberam PHAs e 54,5% (18 de 33 pacientes) entre pacientes que receberam PAIs, respectivamente ( *p* = 0,732). Dos 88 pinos, 43,2% (38 de 88 pinos) apresentaram identificação microbiana, sendo isolados 42 patógenos no total. O *Staphylococcus aureus* foi o mais frequente, representando 59,5% (25 dos 42 patógenos). Nas amostras de “melhor aspecto,” a taxa de recuperação microbiana foi significativamente menor nos PHAs do que nos PAIs, 18,2% (2 patógenos em 11 pinos) e 45,5% (15 patógenos em 33 pinos), respectivamente ( *p* = 0,036). Nas amostras de “pior aspecto,” a taxa de recuperação microbiana nos PHAs se nos PAIs foi 27,3% (3 patógenos em 11 pinos) e 54,5% (18 patógenos em 33 pinos), respectivamente ( *p* = 0,036).

**Conclusão:**

As taxas de recuperação microbiana foram menores nos PHAs comparadas às dos PAIs. Entretanto, estas diferenças não impactaram nas taxas de infecção clínica, que foram semelhantes nos dois grupos.

## Abstract (en)

**Objective:**

To compare the microbial retrieval rates and the organism types on the surface of stainless-steel pins (SSPs) and hydroxyapatite-coated pins (HCPs) from external fixators (EFs).

**Methods:**

The present prospective, non-randomized, multicenter, comparative interventional cohort study occurred from April 2018 to October 2021. The sample consisted of 44 patients with EFs, including 33 with SSPs and 11 with HCPs. We collected two pins from each patient, the one with the best and the one with the worst clinical appearance according to the Maz-Oxford-Nuffield (MON) classification, in an aseptic manner, and sent them for microbiological analysis.

**Results:**

The overall superficial infection (SI) rate was 52.3% (23 of 44 patients), affecting 45.5% (5 of 11) patients with HCPs and 54.5% (18 of 33) patients with SSPs ( *p* = 0.732). Of the 88 pins, 43.2% (38 of 88 pins) yielded microbial identification, with 42 pathogens isolated. *Staphylococcus aureus* was the most frequent organism, accounting for 59.5% (25 of 42 pathogens) of the positive samples. In the best-looking pins, the microbial retrieval rate was significantly lower in HCPs than SSPs, with 18.2% (2 pathogens in 11 pins) and 45.5% (15 pathogens in 33 pins), respectively ( *p* = 0.036). In the worst-looking pins, the microbial retrieval rate in HCPs and SSPs was 27.3% (3 pathogens in 11 pins) and 54.5% (18 pathogens in 33 pins), respectively ( *p* = 0.036).

**Conclusion:**

Microbial retrieval rates were lower in HCPs than in SSPs. However, these differences did not impact clinical infection rates, which were similar in both groups.

## Introdução

O método de fixação externa é amplamente difundido no meio ortopédico para o tratamento de fraturas, pseudoartrose e a correção de deformidades. Define-se por fixador externo (FE) um grupo de aparelhos metálicos que permite manter rigidez ou estabilidade da estrutura óssea através de fios ou pinos transósseos aplicados de forma percutânea. Geralmente são feitos de aço inoxidável, titânio ou ligas metálicas, devido à resistência mecânica desses materiais e à sua capacidade de suportar cargas durante o processo de cicatrização óssea. Além do material de base, a cobertura de superfícies com outros compostos, como hidroxiapatita ou prata, pode melhorar o desempenho do fixador. Apesar de todas as possibilidades de uso, os FEs estão sujeitos a complicações que incluem infecção da ferida operatória, soltura dos pinos e infecção do trajeto dos pinos. 1 2 3

Dentre as complicações, a mais comum é a infecção do sítio de inserção do pino, principalmente em FEs usados por tempo prolongado, com uma incidência variando entre 11,3 e 100% dos casos. 4 5 Atualmente, a infecção do trajeto é definida pela presença de sinais flogísticos ao redor dos pinos, que requerem administração de antibiótico, remoção do fio ou pino, ou até desbridamento cirúrgico. 6 Diversas hipóteses foram propostas para descrever a fisiopatologia, mas é consenso que a inflamação progressiva na presença de microrganismos altera o microambiente tecidual e reduz a capacidade do sistema imune de resistir à proliferação bacteriana. Além disso, as bactérias podem se aderir ao implante e formar biofilme pela produção de matriz extracelular. 4 7

O biofilme é uma estrutura tridimensional, multicelular e metabolicamente menos ativa, que se adere ao osso morto ou ao implante. 8 A formação do biofilme dificulta a ação de antibióticos por restringir a entrada através da matriz extracelular. Além disso, as bactérias associadas a biofilmes apresentam atividade fisiológica diferente daquela de organismos livres, padrão alterado de expressão gênica, crescimento lento estacionário e desenvolvem-se em áreas pobres em oxigênio. 9 Em razão desses mecanismos, para tratar a infecção podem ser necessárias altas doses de antibiótico por período prolongado, associadas a desbridamento ou retirada do implante ortopédico. 8

Na literatura, o *Staphylococcus aureus* é o patógeno mais comumente relatado nas infecções do trajeto do pino, seguido pelo *Staphylococcus epidermidis* , *Pseudomonas aeruginosa* , *Proteus mirabilis* , *Escherichia coli* , *Corynebacterium spp.* , dentre outros. 5 10 11 Logo, havendo necessidade de tratamento com antibiótico empírico, é justificado o uso de drogas anti-estafilocócicas até que se obtenha o resultado da cultura. 5 No entanto, nos casos em que há formação de biofilme, o perfil de ação antimicrobiana pode variar. Em um estudo in vitro, cepas de *S. aureus* resistentes à meticilina (MRSA) sofreram ação bactericida com altas doses de daptomicina em monoterapia, mas apenas a terapia combinada com linezolida foi capaz de sustentar atividade bactericida contra o biofilme da mesma cepa. 12 Em outro estudo, desenvolvido em modelo animal, a formação de biofilme por *P. aeruginosa* foi prevenida com administração de amicacina sistêmica, associada à impregnação dos implantes com claritromicina. 13 Não parece haver correlação entre o quadro clínico e o germe causador da infecção. 10

Dentre as estratégias adotadas para prevenir a infecção, estão descritas algumas como: uso de pinos revestidos ou diferente composição do material, 11 14 15 cuidado diário no entorno dos pinos com remoção das crostas, limpeza com clorexidina, iodo ou soro fisiológico. 4 7 16 17 O revestimento do pino com hidroxiapatita é um dos sistemas mais estudados, com propriedades osteocondutoras e melhora na fixação pino-osso, mas um estudo prospectivo recente não mostrou impacto nas taxas de infecção superficial e profunda associadas aos pinos de FE. 18 Uma metanálise que comparou pinos de aço inoxidável (PAIs) com pinos revestidos de hidroxiapatita (PHAs), prata ou titânio também não evidenciou diferença estatisticamente significativa nas taxas de infecção. 14

Os estudos existentes costumam avaliar e comparar as taxas de infecção nos tecidos superficiais e profundos em contato com os pinos de FE de acordo com o tipo de revestimento utilizado nos pinos; porém, ainda é escassa a literatura para a avaliação da microbiota mais comumente encontrada na superfície desses dispositivos. Este trabalho visa avaliar as taxas de infecção de tecidos em contato com os pinos de FE, descrever os germes que colonizam os pinos de FE, bem como comparar as taxas de recuperação microbiana entre os PAIs e os PHAs.

## Materiais e Métodos

Trata-se de um estudo de coorte prospectiva de intervenção, não randomizado, multicêntrico e comparativo em pacientes submetidos a tratamento cirúrgico com FE de qualquer tipo, no período entre abril de 2018 e outubro de 2021, em 2 hospitais terciários especializados em doenças ortopédicas. Nesses pacientes, foi realizado estudo inicial que comparou as taxas de infecção nos trajetos dos pinos revestidos ou não de hidroxiapatita, 18 e atualmente buscamos comparar as taxas de recuperação microbiana e identificação dos patógenos encontrados nos pinos de FE com e sem revestimento de hidroxiapatita. Esta pesquisa se encontra aprovada no Comitê de Ética em Pesquisa CAAE: 84939418.6.0000.5342 e todos os indivíduos incluídos assinaram o termo de consentimento livre e esclarecido (TCLE).

Foram incluídos os pacientes que concordaram em participar, assinando o termo de consentimento para utilização de dados, e que foram submetidos a tratamento cirúrgico com FE de qualquer tipo, tanto para o tratamento de fraturas quanto para a correção de deformidades, tratamento de osteomielite e/ou pseudoartrose, e com expectativa de manutenção do FE por um período mínimo de três semanas. Os pacientes foram seguidos prospectivamente a cada quatro semanas, ou conforme necessário para o tratamento. Foram excluídos os pacientes que perderam seguimento antes de 1 ano, e os pacientes que permaneceram com FE por menos de três semanas.

Utilizando a classificação de Maz-Oxford-Nuffield (MON) 19 ( Tabela 1 ), previamente validada para infecções associadas aos pinos de FE, coletou-se dois pinos de cada paciente: o que apresentava melhor aspecto clínico nos tecidos ao redor do pino no momento da retirada do FE, e o que apresentava pior aspecto clínico. Assim foram formados dois grupos: um com os pinos de melhor aspecto e um com os pinos de pior aspecto de cada paciente. A taxa de recuperação microbiana foi usada para avaliar a frequência de testes microbiológicos positivos nos pacientes do estudo de uma forma geral. Para calcular a taxa de infecção global, considerou-se infectado o paciente que apresentou qualquer pino com grau 2 ou maior na classificação de MON.

**Tabela 1:** Classificação de Maz-Oxford-Nuffield (MON)

Graus menores	Sinais e sintomas	Tratamento
1. Não visto como clinicamente uma infecção, mas uma reação	Levemente eritematoso, leve descarga	Melhorar cuidados com o pino, observar mudanças no local
2. Clinicamente visto como infecção de pele no local do pino	Eritema da pele, descarga serosa ou purulenta, dor e sensibilidade nos tecidos moles, apto a mobilizar com analgesia.	Melhorar cuidados com o pino. *Swab* para cultura e antibiograma. Início de antibiótico oral anti-S *taphylococcus*
3	Intenso eritema + dor e descarga purulenta com edema. Incapaz de mobilizar com analgesia. Reação periosteal ao rx? Soltura do pino?	Cuidados intensivos no local do pino. Antibiótico IV por 5–7 dias; elevação do membro; Rx para excluir reação periosteal e soltura do pino que, caso presente, torna-se grau 4
**Graus maiores**		
4	Envolvimento grave dos tecidos moles > 1 pino, reação periosteal presente, procurar por osteólise	Sem resposta ao tratamento local ou antibióticos. Remover pino; antibiótico IV por 5–7 dias, plano de colocação de novo pino em novo estágio
5	Envolvimento grave dos tecidos moles > 1 pino + osteomielite	Remover pino: Cirurgia adicional para controle da infecção óssea. Amostras para cultura e antibiograma; antibiótico IV por período prolongado.
6	Sequestro +/− formação de fístula enquanto o tratamento com o aparelho continua	Potencial de remoção do fixador; debridamento do sequestro e coleta de amostras para cultura e antibiograma. Tratamento para salvamento do membro, longo período de antibióticos IV
Seguindo remoção do aparelho 6B	Dor/sensibilidade sobre o antigo trajeto do pino +/− formação de fístula. Sequestro no antigo trajeto do pino ao Rx	Debridamento do trajeto do pino; amostras para cultura e antibiograma; 3 pós-op antibióticos IV; após oral por 2 semanas

**Fig. 1:**
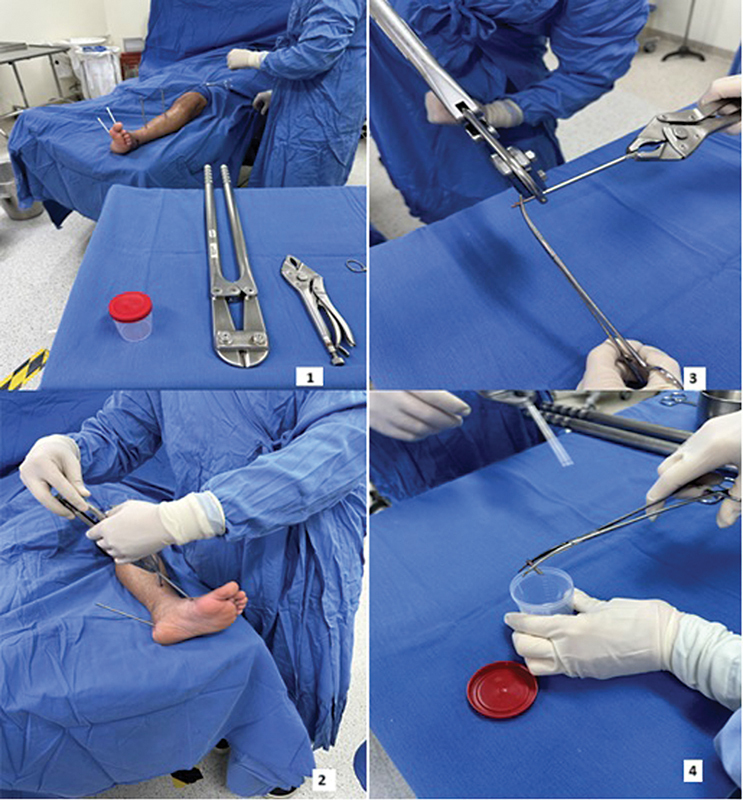
Método de coleta asséptica da ponta intraóssea do pino. ( **1** ) Assepsia e antissepsia com colocação de campos estéreis. ( **2** ) Retirada do pino. ( **3** ) Corte da ponta intraóssea do pino. ( **4** ) Envio da ponta para cultura.

Os pinos foram retirados do FE de forma asséptica, sendo que as pontas intraósseas foram cortadas e enviadas para análise microbiológica em frascos estéreis e devidamente identificados ( Fig. 1 ). As amostras foram homogeneizadas em 3 ml de caldo *brain-heart infusion* (BHI) e inoculadas em ágar sangue aeróbico, ágar chocolate, ágar sangue anaeróbico e caldo de tioglicolato. As placas de ágar sangue e chocolate foram incubadas a 35 a 37°C por 5 dias para culturas aeróbicas, e 14 dias para anaeróbicas. O caldo de tioglicolato foi incubado por 14 dias e, em caso de crescimento bacteriano, o líquido foi semeado em placas de ágar sangue (culturas aeróbicas e anaeróbicas). As colônias bacterianas isoladas foram identificadas por espectrometria de massa. O perfil de sensibilidade foi determinado para todas as cepas identificadas de acordo com técnicas microbiológicas padronizadas pelo *Clinical and Laboratory Standards Institute* . 20

A análise estatística foi realizada utilizando-se o IBM SPSS Statistics for Windows (IBM Corporation) versão 27.0. As variáveis categóricas foram expressas como frequências absoluta e relativa. As associações entre elas foram avaliadas utilizando-se o teste qui-quadrado ou exato de Fischer quando necessário. Considerou-se estatisticamente significativos valores de probabilidade < 0,05.

## Resultados

De todos os pacientes ( *n* = 44) incluídos neste estudo, 33 foram tratados com FE usando PAIs e 11 com PHAs. Da população estudada, 30 (68,2%) eram do sexo masculino e 14 (31,8%) do sexo feminino. Considerando a classificação de MON, o grau máximo encontrado nesta amostragem foi grau 3 ( Tabela 2 ). A taxa de infecção global foi de 52,3% (23 de 44 pacientes), sendo que entre os pacientes com PHAs foi de 45,5% (5 de 11 pacientes) e os pacientes com PAIs foi de 54,5% (18 de 33 pacientes), *p* = 0,732.

**Tabela 2:** Características da população de estudo ( *n* = 44)

	Hidroxiapatita ( *n* = 11)	Aço inoxidável ( *n* = 33)	*p*
**Sexo masculino**	7 (63,6%)	23 (69,7%)	0,722
*Tabagismo*	—	5 (15,2%)	0,309
*HAS*	1 (9,1%)	6 (18,2%)	0,659
*DM*	—	2 (6,1%)	1,000
**Grau máximo na classificação de Maz-Oxford-Nuffield**			
* 1*	6 (54,5%)	15 (45,4%)	0,871
* 2*	2 (18,2%)	7 (21,2%)	
* 3*	3 (27,2%)	11 (33,3%)	
**Taxa de recuperação microbiana**			
* Total de pacientes*	3 (27,3%)	21 (63,4%)	0,036
* Pino de pior aspecto*	3 (27,3%)	18 (54,5%)	0,036
* Pino de melhor aspecto*	2 (18,2%)	15 (45,5%)	0,036

**Notas** : Valores representam frequência absoluta e relativa; *p* , valor de probabilidade (teste do Qui-quadrado).

No total, foram realizadas culturas de 88 pinos (2 de cada paciente), o de melhore o de pior aspecto clínico, de acordo com a classificação de MON. Dos 88 pinos avaliados, em 38 (43,2%) houve recuperação bacteriana, sendo isolados 42 patógenos no total. Dentre os 42 patógenos, o *S. aureus* apareceu 25 (59,5%) vezes, tendo sido o mais frequente.

Dos 11 pacientes que receberam PHAs, 3 (27,3%) apresentaram recuperação microbiana. Destes 3 pacientes, 2 (66,6%) apresentaram bactérias Gram-positivas, 1 (33,3%) apresentou bactéria Gram-negativa e 1 (33,3%) apresentou bactéria multirresistente ( Tabelas 3 , 4 ). Dos 33 pacientes que receberam PAIs, 21 (63,6%) apresentaram recuperação microbiana. Destes 21 pacientes, 16 (76,2%) apresentaram bactérias Gram-positivas, 6 (28,6%) apresentaram bactérias Gram-negativas e 9 (42,3%) apresentaram bactérias multirresistentes ( Tabelas 3 , 4 ). Observou-se que a taxa de recuperação microbiana foi significativamente maior naqueles pacientes que receberam PAIs em relação aos que receberam PHAs, 63,4% (21 de 33 pacientes) versus 27,3% (3 de 11 pacientes), *p* = 0,036.

**Tabela 3:** Proporção de achados de germes Gram-positivos, Gram-negativos e multirresistentes entre pacientes colonizados

	Hidroxiapatita		*p*
	Sim	Não	
**Pino de pior aspecto**	N = 3	N = 21	
Gram-positivo	2 (66,7%)	16 (88,9%)	0,489
Gram-negativo	1 (33,3%)	5 (27,8%)	1,000
MR	1 (33,3%)	9 (44,4%)	1,000
**Pino de melhor aspecto**	N = 3	N = 14	
Gram-positivo	3 (100%)	12 (80,0%)	1,000
Gram-negativo	0	3 (20,0%)	1,000
Multirresistente	0	7 (46,7%)	0,485

**Nota** : Valores expressam frequência absoluta e relativa.

**Tabela 4:** Proporção de achados de germes Gram + , Gram- e multirresistentes entre todos os pacientes

	Hidroxiapatita		*p*
	Sim ( *n* = 11)	Não ( *n* = 33)	
**Pino de pior aspecto**			
Gram-positivo	2 (18,2%)	15 (45,5%)	0,158
Gram-negativo	1 (9,1%)	5 (15,2%)	1,000
Multirresistente	1 (9,1%)	8 (24,2%)	0,411
**Pino de melhor aspecto**			
Gram-positivo	2 (18,2%)	12 (36,4%)	0,456
Gram-negativo	0	3 (9,1%)	0,561
Multirresistente	0	7 (21,2%)	0,165

**Nota** : Valores expressam frequência absoluta e relativa.

Analisando a taxa de recuperação microbiana a partir dos 44 pinos de melhor aspecto clínico, sendo 33 PAIs e 11 PHAs, observou-se recuperação microbiana em 15 PAIs (45,5%) e em 2 PHAs (18,2%). No total, 17 (38,6%) dos 44 pinos de melhor aspecto apresentaram recuperação microbiana ( Tabela 2 ). Analisando a taxa de recuperação microbiana a partir dos 44 pinos de pior aspecto clínico, sendo 33 PAIs e 11 PHAs, observou-se recuperação microbiana em 18 PAIs (54,5%) e em 3 PHAs (27,3%). No total, 21 (47,4%) dos 44 pinos de pior aspecto apresentaram recuperação microbiana ( Tabela 2 ). Observou-se que a taxa de recuperação microbiana em pinos sem revestimento é significativamente maior que aqueles revestidos por hidroxiapatita, e isso foi evidenciado tanto na amostra de pinos de melhor aspecto, respectivamente 45,5% (15 de 33 PAI) versus 18,2% (2 de 11 PHA), *p* = 0,036, quanto nos pinos de pior aspecto, respectivamente 54,5% (18 de 33 PAIs) versus 27,3% (3 de 11 PHAs), *p* = 0,036 ( Tabela 2 ). Dos 21 pacientes com recuperação microbiana nos pinos de pior aspecto, 14 (66,7%) também tiveram cultura positiva nos pinos de melhor aspecto. Dos 17 pacientes com cultura positiva no pino de melhor aspecto, 14 (82,4%) também tiveram cultura positiva no pino de melhor aspecto.

Das bactérias encontradas nas culturas realizadas no seguimento, a que tem maior frequência absoluta é o *S. aureus,* sendo encontrada em 14 amostras de pinos de pior aspecto e em 11 amostras de pinos de melhor aspecto. Em seguida aparecem na mesma frequência a *Serratia marcescens* , encontrada em 2 amostras de pinos de melhor aspecto e 1 de pior aspecto, e o *Enterococcus faecalis,* encontrado em 2 amostras de pinos de pior aspecto e 1 de melhor aspecto. Na sequência aparecem *Acinetobacter baumannii* , *Staphylococcus* coagulase-negativa e outras bactérias descritas na Tabela 5 . Curiosamente, dois pacientes sem crescimento no pino de pior aspecto tiveram crescimento de *S. epidermidis* e *Staphylococcus hominis* no pino de melhor aspecto.

**Tabela 5:** Bactérias identificadas em cultura durante o seguimento e respectivas frequências absolutas

	Pino de pior aspecto	Pino de melhor aspecto
*Acinetobacter baumannii*	1	1
*Bacillus sp.*	1	—
*Enterobacter cloacae*	1	—
*Enterococcus faecalis*	2	1
*Pseudomonas aeruginosa*	1	—
*Staphylococcus aureus*	14	11
*Staphylococcus epidermidis*	—	1
*Staphylococcus hominis*	—	1
*Serratia marcescens*	1	2
*Sphingomonas paucimobilis*	1	—
*Staphylococcus coagulase negativa*	1	1
*Stenotrophomonas maltophilia*	1	—

**Nota** : Valores expressam frequência absoluta.

Observou-se que a realização de cultura proporcionou informações clinicamente relevantes para além da classificação MON. Conforme descrito na Tabela 6 , cerca de 1 em cada 5 pacientes classificados como MON 1, ou seja, sem infecção clínica, apresentaram culturas positivas. Além disso, cerca de metade dos pacientes classificados como MON 2 e 3, ou seja, com infecção clínica, apresentaram culturas negativas.

**Tabela 6:** Prevalência de recuperação microbiana de acordo com a classificação MON ( *n* = 44 pacientes / 88 pinos)

	Grau MON			*p*
	1 *(n = 21/42)*	2 *(n = 9/18)*	3 *(n = 14/28)*	
Pino de pior aspecto	5 (23,8%)	7 (77,8%)	9 (64,3%)	0,014
Pino de melhor aspecto	4 (19,0%)	3 (33,2%)	10 (71,4%)	0,024

**Notas** : Valores expressam frequências absoluta e relativa. *p* , valor de probabilidade (teste do Qui-quadrado).

## Discussão

Neste estudo, as taxas de recuperação microbiana em PHAs foram significativamente menores comparadas as de PAIs. No entanto, não encontramos diferença estatística nas taxas de infecção clínica, conforme Stoffel et al. 14 18 Pizà et al. 11 também não encontraram diferença significativa nas taxas de infecção comparando o trajeto de pinos de PHAs e PAIs, porém demonstraram que pinos revestidos por hidroxiapatita apresentam maior adesão pino-osso e melhor osteointegração, levando a menores índices de soltura dos pinos. Pieske et al. 21 concluíram que a melhor adesão pino-osso encontrada nos PHAs é clinicamente irrelevante por não diminuir as taxas de afrouxamento do pino ou de infecção.

Em relação à taxa de recuperação microbiana, nossos resultados divergem daqueles usualmente encontrados na literatura. Os estudos in vivo são escassos, porém diversos estudos in vitro concluem que a hidroxiapatita é mais propensa à adesão microbiana e formação de biofilme, devido a sua superfície áspera e porosa, que apresenta mais locais de ligação para os germes. McEvoy et al. 22 encontraram uma propensão discretamente maior à formação de biofilme de *P. mirabilis* e *S. epidermidis* em fios de Kirschner revestidos por hidroxiapatita em comparação com aço inoxidável. Resultado semelhante foi encontrado por Oga et al., 23 que avaliaram por microscopia eletrônica discos de diferentes materiais (aço inoxidável, liga de titânio, hidroxiapatita) e demonstraram maior adesão de *S. epidermidis* em discos revestidos por hidroxiapatita em relação ao aço inoxidável. Ravn et al. 24 fizeram análise micro calorimétrica de biofilmes coletados de materiais com superfície lisa (cromo-cobalto, titânio), porosa (hidroxiapatita) e polietileno, sendo que o grupo de superfície porosa apresentou o maior crescimento de biofilme em relação ao demais. Contudo, Arciola et al. 25 encontraram adesão bacteriana significativamente menor de *S. epidermidis* aos PHA em estudo in vitro *,* em que os pinos foram expostos a soluções bacterianas e logo após incubados em meio de cultura. Os autores identificaram que, em solução salina, a hidroxiapatita libera íons de cálcio e fósforo progressivamente no decorrer do tempo, e essa liberação coincide com a diminuição da aderência bacteriana.

Em raro estudo in vivo, 11 investigou-se a taxa de infecção entre PHA e PAI, e não foi encontrada diferença estatística tanto na taxa de infecção quanto na taxa de recuperação microbiana, à exceção da *P. aeruginosa* que foi mais isolada em PHAs, sem encontrar explicação para o achado. Em nosso estudo, a *P. aeruginosa* foi isolada em apenas uma amostra, também em um PHA.

O perfil microbiológico identificado no presente estudo corrobora os achados de outros estudos envolvendo fixadores externos. A bactéria mais identificada nas culturas foi o *S. aureus* , representando cerca de 60% da recuperação microbiana. Na sequência de germes mais prevalentes, observamos *Serratia mercescens* , *E. faecalis* e *A. baumannii* . Pizà et al. 11 identificaram o *S. aureus* como agente etiológico da infecção do trajeto do pino em mais de 50% das vezes, seguido por germes Gram-negativos como *P. aeruginosa* , *P. mirabilis* e *E. coli* . Em um estudo 26 com 106 pacientes na Suécia, ao final de tratamento prolongado com FE, foram realizadas culturas de todas as pontas dos pinos e encontraram 39% de culturas positivas, sendo que o *S. aureus* foi responsável por 63% destas.

Dentre as limitações deste estudo, destacamos a amostragem relativamente pequena de pinos, a possível interferência do uso de antibiótico em pacientes com FE por tempo prolongado, a não diferenciação entre pacientes tratando infecção, fratura ou deformidade, e o fato de não ter sido feita análise mediana de tempo. No entanto, sabe-se que os PHAs não são utilizados usualmente em controle de dano ortopédico.

## Conclusão

As menores taxas de recuperação microbiana nos PHAs comparada com os PAIs, nos dois grupos avaliados, poderiam sugerir maior resistência à adesão microbiana em PHAs. Entretanto, para isso ser confirmado necessitaríamos realizar a sonicação dos implantes extraídos, o que não foi possível ser feito. No entanto, estas diferenças não impactaram nas taxas de infecção clínica, que foram semelhantes nos pacientes que utilizaram FE com pinos com ou sem revestimento. O *S. aureus* foi responsável pela maior parte das culturas positivas.
